# The analgesic efficacy of subcostal transversus abdominis plane block with Mercedes incision

**DOI:** 10.1186/s12871-018-0499-3

**Published:** 2018-04-10

**Authors:** Jian-guo Guo, Hui-ling Li, Qing-qing Pei, Zhi-ying Feng

**Affiliations:** 1 Department of Anesthesiology and Pain Medicine, the First Affiliated Hospital, Zhejiang University School of Medicine, 79 Qing Chun Road, Hangzhou, 310003 People’s Republic of China; 2 Department of Anesthesiology and Pain Medicine, the First People’s Hospital of Beilun District, Ningbo, China

**Keywords:** Postoperative pain, Transverses abdominis plane block, Liver resection, Ropivacaine

## Abstract

**Background:**

Conventional perioperative analgesic modalities (e.g. opioids, epidural analgesia) have their own drawbacks, which limit their clinical application. This study investigated the opioid-sparing effectsof the oblique subcostal transversus abdominis plane (OSTAP) blockade with ropivacaine for the patients undergoing open liver resection with a Mercedes incision.

**Methods:**

126 patients who were scheduled for open liver resection were enrolled in this study. Patients were randomly assigned to receive bilateral ultrasound-guided OSTAPblocks with either 0.375% ropivacaine (groupT) or 0.9% isotonic saline (group C). Both groups also received intravenous patient-controlled analgesia and intravenous 40 mg parecoxib every 12 h for a total of 3 days. Preoperative and intraoperative parameters, plus intraoperative and postoperative cumulative sufentanil consumption, were recorded.

**Results:**

70 patients were enrolled in the study finally. There were no significant differences between the two groups with respect to preoperative parameters, and surgical and anesthetic characteristics. The intraoperative sufentanil use, cumulative sufentanil consumption at 5 min after extubation, 2 h, 4 h,12 h and 24 h after operation in group T was significantly less than that in group C (*P* = 0.001, 0.001, 0.000, 0.000, 0.001 and 0.044, respectively). Compared with group C, postoperative NRS pain scores at rest were significantly lower at 2 h and 4 h postoperatively in group T (*P* = 0.04and 0.02, respectively); NRS scores at the time of coughing were also significantly lower in group T than in group C at all time points except 5 min after extubation (all *P* < 0.001). Furthermore, compared with group C, the number of intraoperative vasodilator use, the extubation time and the incidence of nausea was reduced in group T.

**Conclusion:**

Ultrasound-guided OSTAP block with ropivacaine can significantly decrease the perioperative cumulative dosage of analgesics and improve analgesic effect without obvious side effects for the patients who underwent an open liver resection with Mercedes incision when compared tothe ultrasound-guided OSTAP block with saline.

**Trial registration:**

The study protocol was registered at http://www.chictr.org.cn (ChiCTR-TRC- 14004827) on February 19, 2014.

## Background

Pain control is a vital component to achieve enhanced recovery after liver surgery. Effective postoperative pain control will reduce the incidenceof numerous postoperative complications, can facilitateearly mobilization and may result in earlier recovery [1]. However, optimal postoperative analgesic modalities following liver resection remain controversial [1, 2].Paincontrol is historically achieved by the administration of opioids, whichis associated with well-documented side effects, such as sedation, respiratory depression,pruritus, hallucinations and postoperative nausea and vomiting (PONV). Epidural analgesia, another routinely used analgesic technique, offers equivalent or superior pain scores when compared toconventional systemic opioids. However, its utilizationis limited by perioperative coagulation dysfunction, which is typical in the patients for liver surgery and subsequent catastrophic neurologic injuries resulting from epidural haematoma [3–5]. In addition, epidural analgesia is independently associated with an increased use of blood transfusions and a longer hospital stay [5]. Enhanced recovery following hepatectomy has gained attention even though there is limited evidence on the efficacy and effectiveness of existing analgesic techniques. The oblique subcostal transversus abdominis plane (OSTAP) block has been demonstrated to improve pain-related outcomes after upper abdominal surgeries, such as laparoscopic cholecystectomy [2–4, 6–9]. To our knowledge, the analgesic efficacy of ultrasound-guided OSTAP blocks has not been extensively investigated in the setting of open liver surgery for patients suffering from liver cancer, especially for the roof-shaped incision, including the Mercedes incision [3, 6, 9, 10]. Therefore, we tested the hypothesis that ultrasound-guided OSTAP blocks can reduce cumulativeopioid consumption for the patients undergoing liver resection with the Mercedes incision when added to conventional multi-model intravenous analgesic technique. The aim of this prospective comparative investigation was to assess the impact of OSTAP blockade in a multimodal perioperative analgesic regimen and any related side effects in patients undergoing liver resection with Mercedes incision.

## Methods

This prospective,observer blinded randomized control trialwas conducted at the First Affiliated Hospital, School of Medicine, Zhejiang University in China after being approved by the Ethical Committee of Zhejiang University (Hangzhou, People’s Republic of China) (Ref: 2013–662). Each patient read and signed a consent form before enrolment in the study.

### Participants

From February 20,2014 to February19, 2015, 126 patients who suffered from hepatocellular carcinoma and scheduled for open liver resection were enrolled in this investigation. Inclusion criteria for open liver resection surgery included tumor size < 10 cm; no intrahepatic or distant metastasis; no invasion of the diaphragm or surrounding tissues; indocyanine green retention rate at 15 min < 15%; a remnant liver volume/standard liver volume ratio of > 50% in patients with liver cirrhosis and > 35% in patients without liver cirrhosis; and Class A or B on the Child–Pugh liver function scale. Exclusion criteria were as follows: the American Society of Anesthesiologists classification (ASA) IV or above, body mass index < 18 or > 26 kg/m^2^, age > 65 or < 18 years old, currently taking contraceptives, pregnancy, inability to understand Mandarin, inability to properly describe postoperative pain to investigators (e.g. language barrier, neuropsychiatric disorder), relevant drug allergy, pre-existing neuralgia, history of chronic pain, consumption of opioids within 24 h before surgery, alcohol or drug abuse, contraindications to peripheral nerve block (e.g. allergy to local anesthetics, coagulopathy, local or systemic infection), history of abdominal surgery or trauma, previous liver resection, rupture or bleeding of the tumor, emergency surgery for liver resection, estimated operation time of more than 6 h, intraoperative bleeding more than 500 mL, transplant donor liver resections, and those unable to be extubated postoperatively for any clinical reason.

### Randomizationand blinding

The study was an observer blinded randomized control trial. The patients were randomly assigned to receive bilateral OSTAP blocks with either 40 mL 0.375% ropivacaine (Naropin; AstraZeneca, Sodertalje, Sweden) (group T) or with 40 mL 0.9% isotonic saline (group C). No adjuvant was added to the solutions. Identical boxes containing either isotonic saline or ropivacaine were sealed and marked with the name of the project, the names of the investigators, and consecutive numbers according to a computer-generated block randomization list provided by www.random.org. Study medication was prepared by a designated nurse (LNZ, who did not participate in direct patient care). The nurse (LNZ) opened the box and drew the study medication into identical syringes. Two of the anesthesiologists (JGG, HLL) performed all intraoperative assessments, and two other investigators (SJY, LC) assessed and recorded all the postoperative data.

### Oblique subcostal transversus abdominis plane (OSTAP) block

After sedation with midazolam, the same ultrasound-guided OSTAP block approach was performed bilaterally by one of two clinical investigators (JGG, HLL) in both groups. Ultrasound images were obtained using Sonosite S-Nerve ultrasound machine (Sonosite®, Micromaxx, Bothwell, WA, USA) with the HFL38x broadband linear array probe. The ultrasound probe was obliquely placed on the upper abdominal wall. The neurofascial layer of OSTAP was identified as lying between the rectus abdominis and the transversus abdominis muscle. To perform the blocks, abdominal skin was prepared with 5% povidone iodine solution and covered with sterile drapes. Local infiltration was performed with 1–2 mL of 2% lidocaine at the needle entry site. A 22G, 120 mm Stimuplex D Plus needle (B. Braun, Melsungen AG, Germany) was advanced using an in-plane technique until the tip lay within the neurovascular fascial plane between the rectus abdominis and the transversus abdominis muscle. Following negative aspiration, a test injection with 1 mL of 0.9% normal saline was performed to confirm the needle location. Then 20 mL ropivacaine (group T) or saline (group C) was injected with intermittent aspiration every 3–5 mL while observing the expansion of intermuscular plane on each side.

Sensory change was then assessed bilaterally between the midclavicular line and the midline, starting above dermatome T4 and moving caudally to dermatome L4. A pinprick was tested with a blunt needle and cold with disinfectant swabs at 10, 20 and 30 min after the OSTAP block by one clinical investigator (QQP). If the intended sensational decrease in surgical dermatomes did not occur after 30 min, the patient was regarded as having a failed block and was excluded from further investigation during the analysis and statistic phase.

### Intraoperative anestheticmanagementbefore and after OSTAP placement

All patients received a standardized anesthetic regimen.No additional preoperative medications were administered after block placement. Upon arrival in the operating room, ASA standard monitoring, including electrocardiography, non-invasive blood pressure and pulse oximetry was established. Pre-sedation heart rate (HR), systolic blood pressure (SBP), diastolic blood pressure (DBP) and mean arterial pressures (MAP) were recorded as baseline values. Supplemental oxygen (oronasal mask at 4 L/min) was administered. After placement of a peripheral venous catheter, a lactated Ringer’s infusion was started at a maintenance rate. With proper sedation an OSTAP block was performed in both groups as stated above.

About 30 min after the OSTAP block, general anesthesia was induced with propofol (0.5–2.0 mg/kg), sufentanil (0.2–0.6 μg/kg) and rocuronium (0.6–1.0 mg/kg). After endotracheal intubation, ventilation was started with oxygen and medical air (FiO_2_ = 0.6) and ventilator settings were adjusted to maintain normocapnia. A total intravenous anesthesia technique composed of propofol, sufentanil, dexmedetomidine and intermittent rocuronium was used to maintain adequate anesthesia with bispectral index (BIS) at 40–50 (A-2000 BIS™ monitor; Aspect® Medical Systems, Inc., Natick, MA, USA). The dexmedetomidine was administrated intravenously with a loading dose of 0.5–0.7 μg/kg/h for 10 min followed by a continuous infusion of 0.2 μg/kg/h until 1–1.5 h before abdomen closure. The propofol effect-site target concentrations were titrated to keep BIS between 40 and 50. If MAP or HR of the patient increased up to 20% of the initial value, intravenous sufentanil 0.05 μg/kg was administered. Repeated doses of sufentanil were given every 5 min to keep the blood pressure around the patient’s baseline values. The amount of sufentanil required was documented. If the MAP or HR of the patient decreased up to 20% of the initial value, the propofol effect-site target concentrations were adjusted first; if BIS was in the range of 40–50, ephedrine 5 mg intravenously was administered, and additional doses of ephedrine were permitted every 2 min to maintain haemodynamic stability. The patient’s nasopharyngeal temperature was maintained between 36.0 °C and 36.5 °C by WarmTouch™(Covidien, Bishop’s Stortford, UK). An independent anesthesiologist (QQP), who wasblinded to the mode of perioperative analgesia, would then complete the recording of the haemodynamic parameters after the bilateral OSTAP blocks.

A Mercedes incision was made about 40 min after the bilateral OSTAP blocks. The subsequent surgical procedure was performed according to the institutional standards by four surgical groups. At the end of the surgeries, these patients were then transferred to the post-anesthetic care unit (PACU). Each patient’s trachea was extubated when extubation criteria, including a response to verbal commands, a spontaneous respiratory rate exceeding 12 breaths/min, Vt more than 5 mL/kg, end-tidal carbon dioxide partial pressure < 45 mmHg, and SPO_2_ more than 92%, were met.

### Postoperative analgesia and antiemetic use

Before the day of the operation, all patients were familiarized with the verbal numerical rating scale (NRS) evaluation ranging from 0 (no pain) to 10 (the worst imaginable pain). A standardized postoperative analgesic regimen was used, which consisted of 40 mg parecoxib (diluted in 4 mL NS) every 12 h initiated 30 min before surgery intravenously for a total of 3 days unless contraindicated due to renal insufficiency or coronary heart disease, etc. The patient controlled analgesia (PCA) device (a PCA pump; GemStar®, Hospira Inc. Lake Forest, IL, USA) was connected at the end of surgery and was set to deliver a bolus of 2 μg of sufentanil with a lockout time of 10 min with a continuous infusion at 1 μg/h. Local anesthetics were not infiltrated into the surgical wound intraoperatively. All patients received antibiotic and regular ondansetron 4 mg per day for PONV prophylaxis until 3 days postoperatively.

Upon extubation, the pain score at rest and on coughing were evaluated by nurses (SJY and LC) who were blinded to the groups using NRS (0–10). All patients received intravenous sufentanil (2 μg) titration from the PCA device bolus at 10 min intervals if their NRS scores were > 3 in PACU. An acute pain service team was responsible for maintenance of the PCA pump according to the hospital standard. Transition from PACU to the surgical ward was considered safe when the patient had achieved > 9 in the modified Aldrete score for at least 10 min. In the ward, pain at rest and on coughing was recorded for each patient using NRS (0–10) at 2, 4, 12, 24 and 48 h after the operation by the research staff. Rescue opioid with intravenous sufentanil was provided by PCA pump bolus when needed. If the patients had inadequate analgesia after 3–5 bolus doses of sufentanil, the acute pain service (APS) staff would increase the bolus dose by 20–25%. If the patient was not satisfied by sufentanil PCA, intramuscular tramadol was administered for breakthrough pain by physicians on the ward. Sedation levels were recorded using the Ramsay Sedation Scale. Excessive sedation was defined as a Ramsay Sedation Scale value of 5 or 6, which required the administration of naloxone.

### Data collection

Preoperative and intraoperative variables included age, gender, weight, height, ASA physical status, liver function tests; estimated intraoperative blood loss (mL); operative time (min); anesthesia time (defined as the time spent in the operating room in minutes); dosage of intraoperative sufentanil administered. Postoperative variables included NRS scores, cumulative sufentanil consumption, the incidence of PONV. Ramsay Sedation Scale scores [11] collected 5 min after extubation, and 2 h, 4 h, 12 h and 24 h after the operation was evaluated as: [1] the patient is anxious and agitated or restless, or both; [2] the patient is co-operative, oriented and tranquil; [3] the patient responds to commands only; [4] the patient exhibits brisk response to light glabellar tap or loud auditory stimulus; [5] the patient exhibits a sluggish response to light glabellar tap or loud auditory stimulus; and [6] the patient exhibits no response.

Dermatome sensory distribution was recorded 30 min after the OSTAP blockade. The incidence of accidental vascular or intraperitoneal puncture, and symptoms suggestive of local anesthetic toxicity were also recorded. All patients were interviewed until 48 h postoperatively. Complications, such as bruises and swelling at the block site, were followed up and managed by APS until completely resolved. The haemodynamic parameters were recorded after the OSTAP blockade at the following time points: T_0_, just before the OSTAP blockade; T_1_, 5 min after the OSTAP blockade; T_2_, 10 min after the OSTAP blockade; T_3_, 20 min after the OSTAP blockade; T_4_, 30 min after the OSTAP blockade, T_5_, just before incision; T_6_, 3 min after incision; T_7_, 5 min after incision; T_8_, 10 min after laparotomy; T_9_, 30 min after laparotomy; and T_10_, 48 h after the operation.

### Sample size

The primary outcome was cumulative sufentanil consumption24h after surgery(intraoperative sufentanilconsumptionand 24 h after operationsufentanilconsumption)in this study. The secondary outcome included the time required to extubate, postoperative NRS scores, side effects, and haemodynamic variations perioperatively. In our preliminary trial of 18 patients, the cumulative sufentanil consumptionwere 89.45 ± 15.5 μg and 80.35 ± 12.89 μg in group C and in group T within 24 h after surgery, respectively. According to the preliminary study, we calculated that 32 patients would be required in each group for a 90% power to detect 10% reduction of cumulativesufentanil consumption at the α level of 0.05. To account for any patient dropouts or missing data, we planned to enroll 40 patients per study group.

### Statistical analysis

Data were analyzedusing SPSS 19.0 software (SPSS, Inc., Chicago, IL, USA). The Kolmogorov-Smirnov test was used to check for normal distribution. Parametric data were expressed as mean with95% confidence intervalsand nonparametric data as median with interquartile range. Group means were compared using the Student’s t-test or the Mann–Whitney U test as appropriate. For continuous data, overall differences were tested by analysis of variance (ANOVA) followed by a post hoc test with least significant difference t-test (LSD) or Kruskal-Wallis test followed by a Mann–Whitney U test when appropriate. Categorical variables were presented as values and percentages and were compared using the χ2 test. Significance was defined as *P* < 0.05.

## Results

**Fig. 1:**
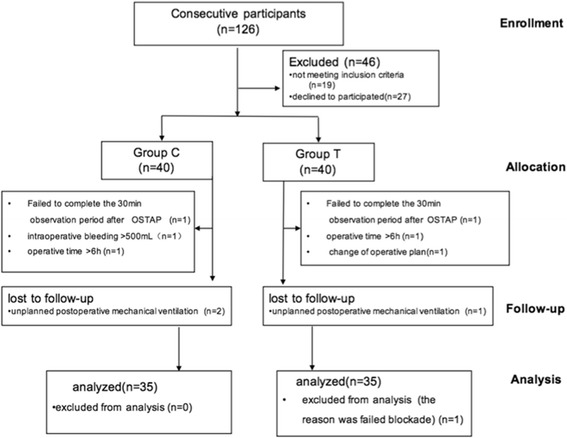
Flow diagram of enrolment

A total of 126 patients were assessed for eligibility; 19 did not meet the inclusion criteria; 27 refused to participate. A total of 10 patients were excluded from the final analysis because of a change of operative plan (*n* = 1); failure to complete the 30 min observation period after OSTAP (*n* = 2); intraoperative bleeding > 500 mL(n = 1); operative time > 6 h (n = 2); unplanned postoperative mechanical ventilation (*n* = 3); and failed blockade in group T (n = 1). Data were analyzedon 35 patients in each group (Fig 1).

### Preoperative parameters

The demographics and preoperative details of the remaining 70 patients are presented in Table 1. There were no significant differences between the two groups with respect to age, weight, ASA class, past medical history of diabetes or hypertension, preoperative laboratory studies, including international normalized ratio, prothrombin time, total bilirubin and aspartate aminotransferase.

**Table 1:** Patient demographic characteristics, comorbidity and liver function tests before surgery in Groups C and T

Variables	Group C (*n* = 35)	Group T(n = 35)	Pvalue
Gender (female/male)	12/23	10/25	0.797
Age(y)	52.6(49.7–55.5)	49.1(44.8–53.3)	0.166
Body weight (kg)	62.5(60.0–65.4)	58.9(56.1–61.7)	0.092
Height (cm)	166.5(164.2–169.2)	165.1(163.1–167.2)	0.389
BMI(kg/m2)	23.1(21.9–25.6)	22.2(19.4–23.3)	0.108
Preoperative			
ASA (I/II/III)	6/25/5	5/25/5	0.962
Hypertension	4	2	0.673
Diabetes	2	1	1.000
ALB value (g/L)	39.4(38.1–40.7)	39.7(38.1–41.4)	0.741
AST value(u/L)	24.0(20.0–31.0)	26.0(19.0–41.0)	0.708
TB (μmol/L)	14.5(10.8–18.3)	13.0(10.0–16.0)	0.393
BUN value(mmol/L)	5.2(4.6–5.6)	5.2(4.6–5.7)	0.959
CR (umol/L)	65.2(61.8–70.2)	69.1(64.2–74.0)	0.235
INR value	1.1(1.0–1.2)	1.1(1.0–1.2)	0.640
PT value(s)	12.5(11.8–13.3)	12.9(11.4–13.7)	0.428

Values are expressed as mean (95%confidence interval) for normal distributed variables, median (interquartile range) for skew distribution, and numbers for absolute values

*ALB* Serum albumin, *AST* Aspartate aminotransferase, *BMI* Body mass index, *BUN* Blood urea nitrogen, *CR* Serum creatinine, *INR* International normalized ratio, *PT* Prothrombin time, *SA* American Society of Anesthesiologists, *TB* Total bilirubin

*P < 0.05 compared to the control group

### Intraoperative parameters

Surgical and anesthetic characteristics are shown in Table 2. No significant differences were observed in incision length, time from classic TAP block to incision, operative time, estimated intraoperative blood loss and intravenous cystaloids administered between group C and group T. In addition, no significant difference was found among the five surgical teams or anesthetists between the two groups. In group T, less intraoperative propofol dosage was required compared with group C, but the difference was not statistically significant (Table 2). Compared with group C, the time to extubation was significantly shorter in group T (*P* = 0.02). There were no differences between the two groups regarding the number of patients using ephedrine (vasopressor). However, the number of patients that required a vasodilator intraoperatively in group T was significantly less than that in group C (*P* = 0.002).

**Table 2:** Surgical and anesthetic characteristics in Groups C and T

Variables	Group C (*n* = 35)	Group T (n = 35)	*P* value
Time from TAP to incision(min)	47.0(44.3–50.2)	46.9(43.5–47.8)	0.626
Incision length(cm)	30.0(23.8–35.0)	25.0(20.0–35.0)	0.703
Operation time(min)	193.9(172.7–287.1)	193.8(159.7–261.9)	0.377
Intraoperative intravenous crystalloids	2250(1750–3030)	2250(1750–3120)	0.872
Estimated blood loss (mL)	200.0(100.00–350.0)	200.0(100.0–400.0)	0.345
Urine output(mL)	200.0(150.0–400.0)	200.0(140.0–400.0)	0.894
Intraoperative Propofol(mg)	1168.7(986.0–1681.1)	1121.7(866.1–1291.2)	0.276
Extubation time(min)	59.9(51.7–85.0)	45.35(39.9–61.25)*	0.022
Surgeon			0.097
Group 1	10	10	
Group 2	3	0	
Group 3	11	5	
Group 4	7	13	
Group 5	4	7	
Anesthetist			0.632
Group 1	19	17	
Group 2	16	18	
Intraoperative ephedrine use			1.000
yes(n)	4	5	
No(n)	31	30	
Intraoperative antihypertensives			0.002
yes(n)	24	10*	
No(n)	11	25	

Values are expressed as mean (95%confidence interval) for normal distributed variables, median (interquartile range) for skew distribution, and numbers for absolute values

*TAP* transversus abdominis plane

*P < 0.05 compared with Group C

### OSTAP blockade

**Fig. 2:**
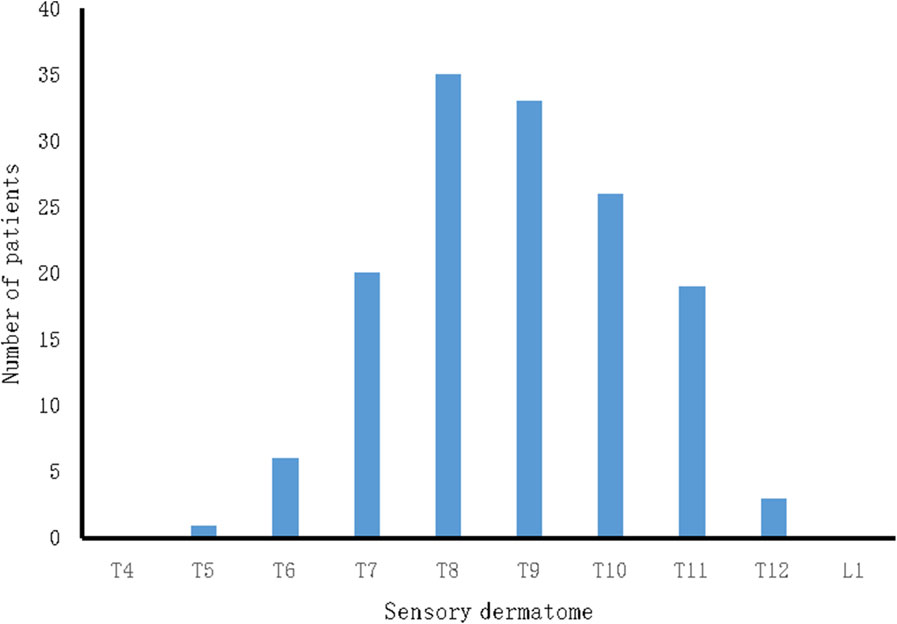
Sensory dermatomal distribution in group T at 30 min after oblique subcostal transversus abdominis plane block

In both groups, the OSTAP was easily performed using ultrasonography, and the procedures were carried out without any complications. During statistics, the effect of OSTAP blockade was checked one by one in both groups. In group C, no patients had the dermatome sensory distribution by pinprick approach. One patient had failed blocks in Group T and was excluded from the final analysis. The OSTAP block in group T resulted in a distribution of sensory block ranging from T5 to T11 (Fig. 2). None of the patients suffered from any complications related to the bilateral OSTAP block, such as vascular or intraperitoneal puncture, local anesthetics systemic toxicity, bruises or swelling at the block site.

### Perioperative pain control

**Fig. 3:**
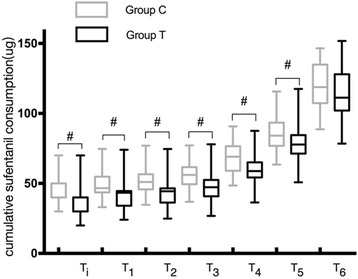
Cumulative sufentanil consumption in Groups C and T. Ti:intraoperative period;T_1_, 5 min afterextubation;T_2_, 2 h after operation; T_3_, 4 h after operation; T_4_, 12 h after operation; T_5_, 24 h after operation;T_6_, 48 h after operation.^#^ P < 0.05 compared with Group C

The intraoperative sufentanil use in group T was significantly less than that in group C (*P* = 0.001). Compared with group C, cumulative sufentanil consumption at 5 min after extubation, and 2 h, 4 h, 12 h and 24 h after the operation was significantly lower in group T (P = 0.001, 0.000, 0.000, 0.001 and 0.044, respectively) (Fig 3). Nevertheless, no significant difference was found in the cumulative sufentanil consumption at 48 h after opertation between the two groups (*P* = 0.181). Following discharge from the recovery unit to the one-day surgery ward, there were no differences between the groups in the requirements for non-opioid analgesic medication. However, four patients in group C and one in group T could not be satisfied by sufentanil PCA and they received intramuscular tramadol 100 mg for breakthrough pain. Group C required more rescue tramadol compared to group T, but the difference was not statisticallysignificant (*P* > 0.05).

Both groups delivered good analgesia at rest. However, compared with group C, the OSTAP block significantly reduced postoperative NRS pain scores at rest, with (median (interquartile range)) at 2 h after the operation (2 [1, 2] vs. 2 [1–3]) and 4 h after the operation (1 [1, 2] vs 2 [1–3]). NRS scores at the time of coughing were also significantly lower in group T than in group C at all time points except 5 min after extubation (all *P* < 0.001) (Table 3).

**Table 3:** Postoperative verbal numerical rating scale (NRS) scorein Groups C and T

Variables	Group C (n = 35)	Group T (n = 35)	P value
NRS score at rest			
T_1_	2(1–4)	2(1–3)	0.070
T_2_	2(1–3)	2(1–2)*	0.044
T_3_	2(1–3)	1(1–2)*	0.024
T_4_	2(1–3)	2(1–3)	0.070
T_5_	1(1–2)^#^	1(1-2)	0.402
T_6_	1(1–2)^#^	1(1-2)^#^	0.336
NRS score at cough			
T_1_	4(2–9)	4(2–7)	0.186
T_2_	4(3–7)	3(1–5)^*#^	< 0.001
T_3_	4(3–8)	3(1–4) ^*#^	< 0.001
T_4_	4(3–7)	2(1–6) ^*#^	< 0.001
T_5_	3(2–6)	2(1–6) ^*#^	< 0.001
T_6_	3(2–6)^#^	2(1-5) ^*#^	< 0.001

Values are expressed as median (interquartile range) for skew distribution. NRS, verbal numerical rating scale; T_1_, 5 min afterextubation;T_2_, 2 h after operation; T_3_, 4 h after operation; T_4_, 12 h after operation; T_5_, 24 h after operation;T_6_, 48 h after operation

^#^*P* < 0.05 compared to T_1_; * *P* < 0.05 compared with Group C

### Nausea, vomiting and sedation

**Fig. 4:**
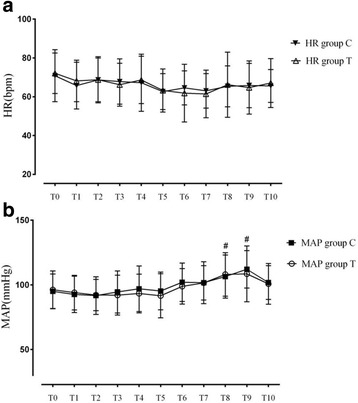
The hemodynamic changes after OSTAP blockage in both groups. **a**: The changes of perioperative heart rate after OSTAP blockage in both groups. **b**: The changes of perioperative mean arterial pressure after OSTAP blockage in both groups. T_0_: just before OSTAP blockage; T_1_: 5 min after OSTAP blockage; T_2_:10 min after OSTAP blockage; T_3_: 20 min after OSTAP blockage; T_4_: 30 min after OSTAP blockage; T_5_: just before incision; T_6_: 3 min after incision; T_7_: 10 min after incision; T_8_: 10 min after laparotomy; T_9_: 15 min after laparotomy;T_10_: 30 min after laparotomy.HR = heart rate; MAP = mean arterial pressure; OSTAP = oblique subcostal transversus abdominis plane. ^#^*P* < 0.05 compared to T_0_; * *P* < 0.05 compared with group C

Compared to group T, group C demonstrated a higher incidence of nausea between 4 h and 8 h after the operation (22.9% vs 5.71%; *P* < 0.05). However, there were no significant differences on the sedation score, the number of times of vomiting and hypotension postoperatively (*P* > 0.05; Fig. 4). The incidence of pruritus was very low, and there was no significant difference between both groups (1 vs. 1 postoperatively; *P* > 0.05) (Table 4).

**Table 4:** Postoperative nausea, vomiting, hypotension and sedation scoresin Groups C and T

Variables	Group C (*n* = 35)	Group T (*n* = 35)	*P* value
Nausea(n)			
T_1_–T_2_	1	0	0.31
T_2_–T_3_	1	2	0.56
T_3_–T_4_	8	2*	0.04
T_4_–T_5_	1	2	0.56
T_5_–T_6_	6	3	0.28
Vomiting(n)			
T_1_–T_2_	1	0	0.31
T_2_–T_3_	1	1	1.00
T_3_–T_4_	4	2	0.39
T_4_–T_5_	1	1	1.00
T_5_–T_6_	1	2	0.56
Hypotension(n)			
T_1_–T_2_	1	0	0.3
T_2_–T_3_	2	2	1.00
T_3_–T_4_	2	1	0.56
T_4_–T_5_	0	0	1.00
T_5_–T_6_	0	1	0.31
RAMSAY score			
T_1_	2.66(2.42–2.89)	2.57(2.35–2.80)	0.594
T_2_	2.14(2.00–2.29)^#^	2.09(1.99-2.18)^#^	0.514
T_3_	2.03(1.97–2.09)^#^	2.03(1.97-2.09)^#^	0.514
T_4_	2.00(1.83–2.06)^#^	1.94(1.83-2.01)^#^	0.321
T_5_	1.97(1.91–2.03)^#^	2.00(2.00-2.00)^#^	0.321
T_6_	1.97(1.91–2.03)^#^	2.00(2.00-2.00)^#^	0.321

Values are expressed as median (interquartile range) for normal distributed variables, median (interquartile range) for skew distribution, and numbers for absolute values.T_1_, 5 min afterextubation;T_2_, 2 h after operation; T_3_, 4 h after operation; T_4_, 12 h after operation; T_5_, 24 h after operation;T_6_, 48 h after operation

^#^*P* < 0.05 compared to T_1_; * *P* < 0.05 compared with Group C

### Hemodynamics

According to the protocol, the hemodynamics parameters were also recorded before and after the OSTAP block and at incision. There were no significant differences between baseline values of HR (T_0_) and at T_1_ through T_10_. In addition, there was no statistically significant difference of HR between the two groups at all time points (*P* > 0.05; Fig. 4). Compared to baseline values, the MAP after laparotomy was significantly higher in both groups (*P* < 0.05; Fig. 3); however, there was no significant difference between the two groups (P > 0.05; Fig. 4). Moreover, there was no significant difference in HR and oxygen saturation by pulse oximetry (Sp0_2_) between the two groups (P > 0.05; data not shown).

### Postoperative complications

No patients suffered from liver dysfunction postoperatively. Postoperative complications included pulmonary infection (four in group C and two in group T occurred 3 days after the operation, P > 0.05) and renal insufficiency (one in group T 2 days postoperatively,P > 0.05). Finally, all the patients were discharged home in a good condition.

## Discussion

To our knowledge, this was the first observe blinded randomized and controlled study to evaluate the use of the OSTAP blockade for patients undergoing an open liver resection with a Mercedes incision. We found that when comparing the control group OSTAP with saline, the ropivacaine OSTAP blockade had improved perioperative analgesia with decreased opioid use both intraoperatively and postoperatively. Furthermore, the ropivacaine OSTAP blockade decreased the intraoperative vasodilator use, the extubation time, the incidence of nausea, and the pain scores, which were reported from 0 to 4 h postoperatively. It demonstrated that the OSTAP blockade with ropivacine offers postoperative analgesic benefit for the Mercedes incision in patients undergoing hepatectomy.

Liver cancer is one of the most common malignancies worldwide, especially in China. Hepatic resection is currently the best choice among all therapeutic strategies. As part of the multimodal approach for perioperative analgesia, classic TAP block has been widely accepted in patients who have undergone lower abdominal surgery, with an evident reduction of the morphine requirement within the first 24 h postoperatively, and a significant decrease of opioid-related side effects, such as PONV [2, 7, 9, 12–14]. There are three common approaches of performing a TAP blockade: subcostal, classic midaxillary and ilioinguinal-iliohypogastric. The distribution of local anesthetic and the extent of sensory blockade differ among these three approaches [9, 12]. For the upper abdomen operation, Hebbard [15] originally described the OSTAP approach in 2008, which was thought to exhibit effective analgesia involving dermatomes T6–T10 (for upper abdominal surgery). Lee et al. [16] found that the subcostal approach may block as cephalad as T8 (interquartile range T7–T9). Recently, the OSTAP block was reported to produce better analgesia than the classic TAP block or intravenous opioid analgesia during the first postoperative 24 h period in patients undergoing laparoscopic cholecystectomy [17]. Based on this investigation, we used the subcostal approach to perform a TAP block for open hepatectomy. Our findings were in agreement with existing investigations on upper abdominal surgery with a bilateral OSTAP blockade [7–9, 18–20].

To our knowledge,there are few reports on the use of the TAP block for major hepatobiliary surgery [3, 6, 10]. Eldin et al. [3] demonstrated that combining postoperative continuous TAP (3 days) and intravenous PCA improved postoperative pain management and reduced fentanyl consumption, with a shorter stay in the intensive care unit for the patient undergoing liver resection with an inverted L-shape incision. The most common approaches for hepatectomy are midline, J-shaped, and Mercedes incisions. Usually, the incision type used depends on the surgeon’s preference and experience. Demirbas et al. [21] concluded that the duration of analgesics use was longer in the J-shaped incision than in the Mercedes incision. Mercedes incisions have been the most frequently used by our liver surgeons for adequate exposure of the liver from surrounding structures. This incision was performed along the path of vessels and nerves, which resulted in lighter tension on the incision and less damage to vessels and nerves of the abdominal wall. Moreover, this incision has been reported to provide faster healing and a better cosmetic outcome.

A recent meta-analysis [8] suggested that the optimal time for TAP block placement should be preoperatively, as opioid requirement and pain score were significantly reduced in comparison with that of TAP block performed postoperatively. In this investigation, we performed the OSTAP block before general anesthesia induction and endotracheal intubation. Our results showed that several intraoperative parameters in group T, including sufentanil dosage, the number of patients who used a vasodilator and the time to extubation, were significantly less than those in group C. Compared with group C, cumulative sufentanil consumption till postoperative24h and NRS pain scores at 2 h and 4 h postopertively decreased significantly in group T. Our results were consistent with previous findings where TAP blocks have been described to last from 6 h to 24 h using ropivacaine [7, 16, 22–24]. The duration of the peripheral nerve block depends on several factors, such as the choice of local anesthetics, the site of injection and the presence of adjuncts. Ropivacaine is the most frequently used local anesthetic for peripheral nerve block in the People’s Republic of China [22]. Lee et al. [16] demonstrated that maximal dermatome spread was observed at 30 min and usually regressed by 24 h. Although few studies have shown the analgesic effect lasting longer than 24 h [25], Stoving et al. [24] demonstrated that the blockade duration of ultrasound-guided unilateral TAP block with 20 mL 75 mg/mL ropivacaine was approximately 10 h with a large variation in healthy volunteers. A meta-analysis about the analgesic efficacy of TAP in 31 controlled trials including 1611 adult participants who underwent abdominal laparotomy, abdominal laparoscopy, or caesarean delivery, showed that pain scores at rest and on movement were reduced at 6 h postoperatively. In addition, TAP block reduced intravenous morphine consumption at 6 h and 12 h postoperatively [7]. Therefore, using a longer-acting local anesthetic, such as liposomal bupivacaine [2, 26, 27] or continuous TAP blocks [2, 10, 28] with catheters may provide extended duration, longer pain control in the postoperative period and, therefore, further decrease the need for postoperative opioids. Randomizedtrials are needed to further investigate the effect of continuous TAP blocks or liposomal bupivacaine on the pain control and the patients’ recovery after hepatectomy.

Tominimizeconfounding factors and increase comparability, we elected to keep the external conditions and procedures identical between the two groups. In this investigation, sensory block was assessed 10 min, 20 min and 30 min after the block was placed, and one patent in group T was excluded from the final analysis because of a failed blockade. Mitchel et al. [19] conducted an experiment in awake volunteers and found that an ultrasound-guided OSTAP blockade can have a dermotomal span up to T4–L4 segments with maximal sensory loss after 30 min. Lee et al. [16] found that the subcostal approach blocked a median of four segments (interquartile range 3–5), with the most cephalad being T8 (interquartile range T7–T9). Maximum dermatomal spread was observed at 30 min. In this study, the OSTAP block in group T resulted in a distribution of sensory block ranging from T5 to T11, which is less than that reported by Wassef et al. [29]. The time for incision in this investigation was 40 min after OSTAP, which was at the peak of the blockade effect. No significant differences were observed in incision length, time from TAP to incision, operative time, estimated total intraoperative blood loss or amount of intravenous fluid administered between group C and group T. The two anesthesiologists performing the OSTAP blocks or intraoperative anesthesia management were blinded to the study; and the nurses who recorded the parameters and the postoperative follow-up were also blinded to this investigation.

One of the main concerns about the OSTAP blockade is the systemic toxicity of the local anesthetics. The study by Griffiths et al. [30]reported potentially toxic ropivacaine concentrations following the use of TAP blocks in gynecologic surgery when a similar total dose of ropivacaine (3 mg/kg) was used. Toju et al. [31] found that the administration of ropivacaine at 3 mg/kg during OSTAP led to rapid increases in plasma concentration during the first 2 h after the blockade and the Cmax nearly reached the threshold for systemic toxicity. Ropivacaine is predominantly eliminated by extensive metabolism in the liver, which depends on hepatic blood flow as well as the degree of protein binding. Edouard et al. [32] reported that the resection of three or more liver segments was associated with a 53% decrease in the free ropivacaine clearance. Therefore, the first limitation was that we did not measure plasma ropivacaine concentrations in group T perioperatively, although none of the patients had symptoms of local anesthetic systemic toxicity (e.g. tinnitus, seizures, cardiovascular collapse) either immediately following the OSTAP blocks or in the PACU postoperatively. In addition, patients in this investigation were not followed up beyond hospital discharge at postoperative day for potential chronic pain. Finally, these data represent the experience at a single academic institution with ASA III or less patients with relatively low BMI, and may not be generalizable to the broader international population.

## Conclusion

The ultrasound-guided OSTAP block has become an important analgesic modality for upper abdominal surgery, such as the hepatectomy with a Mercedes incision. It provids an opioid-sparing analgesic effect without obvious side effects. However, the modest duration of a single shot OSTAP is a significant limitation. OSTAP with continuous catheterization or with local anesthetics of longer duration needs to be further investigated.

## Abbreviations

ASA: American Society of Anesthesiologists classification
DBP: Diastolic blood pressure
HR: Heart rate
MAP: Mean arterial pressures
OSTAP: Oblique subcostal transversus abdominis plane
PACU: Post-anesthetic care unit
PCA: Patient-controlled analgesia
PONV: Postoperative nausea and vomiting
SBP: Systolic blood pressure
